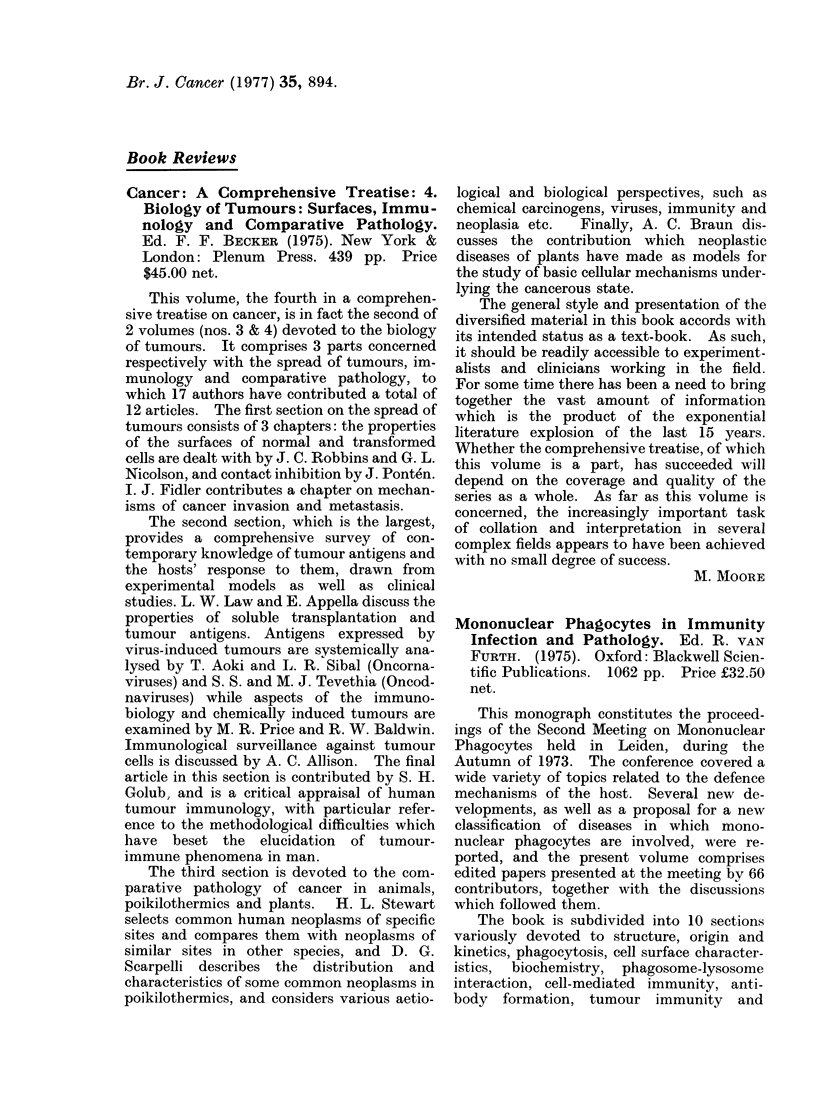# Cancer: A Comprehensive Treatise: 4. Biology of Tumours: Surfaces, Immunology and Comparative Pathology

**Published:** 1977-06

**Authors:** M. Moore


*Br. J. Cancer* (1977) **35**, 894.

### *Book Reviews*

**Cancer: A Comprehensive Treatise: 4. Biology of Tumours: Surfaces, Immunology and Comparative Pathology.** Ed. F. F. Becker (1975). New York & London: Plenum Press. 439 pp. Price $45.00 net.

This volume, the fourth in a comprehensive treatise on cancer, is in fact the second of 2 volumes (nos. 3 & 4) devoted to the biology of tumours. It comprises 3 parts concerned respectively with the spread of tumours, immunology and comparative pathology, to which 17 authors have contributed a total of 12 articles. The first section on the spread of tumours consists of 3 chapters: the properties of the surfaces of normal and transformed cells are dealt with by J. C. Robbins and G. L. Nicolson, and contact inhibition by J. Pontén. I. J. Fidler contributes a chapter on mechanisms of cancer invasion and metastasis.

The second section, which is the largest, provides a comprehensive survey of contemporary knowledge of tumour antigens and the hosts' response to them, drawn from experimental models as well as clinical studies. L. W. Law and E. Appella discuss the properties of soluble transplantation and tumour antigens. Antigens expressed by virus-induced tumours are systemically analysed by T. Aoki and L. R. Sibal (Oncornaviruses) and S. S. and M. J. Tevethia (Oncodnaviruses) while aspects of the immunobiology and chemically induced tumours are examined by M. R. Price and R. W. Baldwin. Immunological surveillance against tumour cells is discussed by A. C. Allison. The final article in this section is contributed by S. H. Golub, and is a critical appraisal of human tumour immunology, with particular reference to the methodological difficulties which have beset the elucidation of tumour-immune phenomena in man.

The third section is devoted to the comparative pathology of cancer in animals, poikilothermics and plants. H. L. Stewart selects common human neoplasms of specific sites and compares them with neoplasms of similar sites in other species, and D. G. Scarpelli describes the distribution and characteristics of some common neoplasms in poikilothermics, and considers various aetiological and biological perspectives, such as chemical carcinogens, viruses, immunity and neoplasia etc. Finally, A. C. Braun discusses the contribution which neoplastic diseases of plants have made as models for the study of basic cellular mechanisms underlying the cancerous state.

The general style and presentation of the diversified material in this book accords with its intended status as a text-book. As such, it should be readily accessible to experimentalists and clinicians working in the field. For some time there has been a need to bring together the vast amount of information which is the product of the exponential literature explosion of the last 15 years. Whether the comprehensive treatise, of which this volume is a part, has succeeded will depend on the coverage and quality of the series as a whole. As far as this volume is concerned, the increasingly important task of collation and interpretation in several complex fields appears to have been achieved with no small degree of success.

M. Moore